# Family history as a risk factor for recurrent hospitalization for lone atrial fibrillation: a nationwide family study in Sweden

**DOI:** 10.1186/1471-2261-12-121

**Published:** 2012-12-10

**Authors:** Bengt Zöller, Henrik Ohlsson, Jan Sundquist, Kristina Sundquist

**Affiliations:** 1 Center for Primary Health Care Research, CRC, Skåne University Hospital, Building 28, Floor 11, Jan Waldenströms gata 35, Malmö, S-205 02, Sweden; 2 Stanford Prevention Research Center, Stanford University School of Medicine, Stanford, California, USA

**Keywords:** Atrial fibrillation, Family history, Risk factors, Genetics

## Abstract

**Background:**

Although the heritability of atrial fibrillation (AF) has been determined, the relevance of family history of AF for the likelihood of recurrent hospitalization for AF is unknown. The aim of this nationwide study was to determine whether family history of AF is a risk factor of recurrent hospitalization for lone AF (LAF), i.e., AF with unknown etiology. The familial risk for first time LAF hospitalization was also determined and compared to the risk of recurrent hospitalization for LAF.

**Methods:**

We examined whether family history of AF is a risk factor for recurrent hospitalization for LAF in the whole Swedish population. We linked Multigeneration Register data on individuals aged 0–60 years to Hospital Discharge Register data for the period 1987–2009 to compare LAF recurrent hospitalization risk among individuals with and without parental or sibling history of AF. We calculated hazard ratios (HRs) to determine the familial HR of recurrent hospitalization for LAF. Odds ratios (OR) were calculated for familial risk of first time LAF hospitalization.

**Results:**

The risk of recurrent LAF hospitalization was 1.23 (95% CI 1.17-1.30) for individuals with affected parents compared to 1.30 (95% CI 1.22-1.38) for those with affected siblings. After 10 years of follow up 50% of those without and 60% of those with family history had recurrent hospitalization for LAF. The risk of recurrent LAF hospitalization in individuals with two affected parents was 1.65 (95% CI 1.44-1.90). There was an interaction between age and family history, with family history having a weaker effect on LAF hospitalization risk in older age groups. The OR for first time LAF hospitalization was 2.08 (95% CI 2.02-2.15) for offspring with affected parents and 3.23 (95% CI 3.08-3.39) for individuals with affected siblings.

**Conclusions:**

Family history of AF is a novel risk factor for recurrent LAF hospitalization. The higher recurrence hospitalization risk in multiplex families and younger individuals suggests a genetic contribution. However, the familial risk for recurrent LAF hospitalization was much lower than the risk for first time LAF hospitalization, suggesting that familial and possibly genetic factors are more important for first time LAF hospitalization than recurrent LAF hospitalization.

## Background

Atrial fibrillation (AF) is a major public health problem because of its increasing prevalence and because it is associated with increased morbidity and mortality [1,2]. Many risk factors for AF have been described and they include, e.g., old age, cardiomyopathy, valvular disease, ischemic heart disease, heart failure, thyroid disease, hypertension and diabetes mellitus [1-3]. In some patients, its etiology remains unknown. AF occurring before the age of 60 years without any evidence of associated cardiopulmonary or other comorbid disease has been termed lone AF (LAF) [4,5]. Familial AF was first reported in 1943 [6], and familial clustering of AF has been repeatedly demonstrated [7-15]. The first chromosomal location of an AF susceptibility gene was reported in 1997 based on genetic mapping studies in three families [16]. Several genetic variants have since then been linked to risk of AF [17-25]. The importance of family history for LAF has been determined in several studies [9,11,12,14].

Despite the use of antiarrhythmic agents for sinus rhythm maintenance in cardioverted AF patients, a considerable proportion of patients relapse to AF [2]. It is generally believed that these recurrences are associated with older age, atrial dilatation, and long duration of AF [26]. Studies have also suggested that female sex, obesity, personal history of two or more AF events, decreased renal function, increased circulating markers of cardiomyocyte injury/strain (high-sensitivity troponin T, N-terminal probrain natriuretic peptide and mid-regional proatrial natriuretic peptide) and endothelin, and increased C-reactive protein to be risk factors for relapse to AF [26-32]. The tendency of AF to become sustained over time is not easily managed, and represents a challenging therapeutic problem. Although family history is a risk factor for first event of AF [6-15], the risk of AF recurrence in patients with a family history of AF has not yet been determined.

In this nationwide study, we investigated the relationship between family history of AF and the risk of recurrent hospitalization for LAF. The familial risk for first LAF hospitalization was also determined and compared to the risk of recurrent hospitalization for LAF.

## Methods

To assess LAF among individuals in Sweden, comprehensive register and health care data from multiple nationwide sources were linked [33-37]. This linking was based on the unique 10-digit personal ID numbers assigned at birth or immigration to all Swedish residents for life, information on which is nearly 100% complete. These numbers were replaced with serial numbers to preserve anonymity. Our database contains data from four sources:

1. The Swedish Multigeneration Register, which contains information on family relationships (siblings, parent-offspring). The register contains information on index persons registered in Sweden between January 1, 1961 and December 31, 2008 and born between January 1, 1932 and December 31, 2008.

2. The Swedish Hospital Discharge Register, which contains information on all hospital diagnoses for all people in Sweden for the period 1987–2009. Each record includes the main discharge diagnosis.

3. The Swedish Cause of Death Register, which contains data on date of death for the period 1961–2010.

4. The Total Population Register, which includes data on year of birth, gender, country of birth and education.

This study was approved by the Ethics Committee of Lund University, Sweden.

### Variable definition

Cases of LAF were identified in the Swedish Hospital Discharge Register by the use of the ICD (International Classification of Diseases) codes 427D (ICD-9) and I48 (ICD-10) and age ≤60 years at the first diagnosis of AF. Individuals with one or more of the following discharge diagnoses within the 5-year period prior to the first diagnosis of AF were excluded: Hypertension (401–405 (ICD-9) and (I10-I15 (ICD-10)); Heart failure (428 (ICD-9) and I50 (ICD-9)); Coronary heart disease (410–414 (ICD-9) and I20-I25 (ICD-9)); Morbi rheumatici cordis and Valvular disease (390–398, 421 and 424 (ICD-9) and I00-I09 and I33-I39 (ICD-10)); Cardiomyopathy (425 (ICD-9) and I42 and I43 (ICD-10)); Myocarditis (422 (ICD-9) and I40 and I41 (ICD-9)); Pericarditis (420 (ICD-9) and I30-I32 (ICD-10)); Other heart disease (429 (ICD-9) and I51 (ICD-10)); Thyrotoxicosis (242 (ICD-9) and E05 (ICD-10)); and Diabetes mellitus (250 (ICD-9) and (E10-E14 (ICD-10)). These diagnoses, together with the same diagnoses from the Cause of Death Register, were also used to define cardiovascular disease (CVD) outcome in model 1.G (see below).

AF in parents and siblings was defined by the ICD codes 427D (ICD-9) and I48 (ICD-10). Parental history of AF was defined as AF in at least one parent during the study period. Sibling history of AF was defined as AF in at least one sibling sometime during the study period.

The validity of the diagnosis of AF has been evaluated, and diagnoses were found to be correct in 97% of cases in the Hospital Discharge Register [37,38]. Diagnoses of other cardiovascular disorders such as stroke and myocardial infarction have an approximate 95% validity [37]. Generally, the validity in the Hospital Discharge Register is approximately 85-95% [37].

### Sample

The analyses were based on a database containing information on all cases of LAF during the period 1987–2009 (n=29,660, mean age=50.1 years (SD=9.6), 74% men, AF recurrence rate=49.6%).

### Statistical methods

We used Cox proportional hazards models in order to investigate recurrence of AF within 10 years. Cases were followed from date of LAF diagnosis during the study period until AF recurrence, death, emigration or the end of the follow-up period (December 31, 2009 or a maximum of 10 years) (whichever came first). In the first analysis, a parent-offspring analysis, we investigated all LAF proband cases whose parents both lived in Sweden sometime between 1987 and 2009 (n=16,160). In model 1.A, parental history of AF was included as a covariate (yes/no), and in model 1.B we also included sex, age at diagnosis of LAF (centered at the mean value), and terms for the interaction between parental history of AF and age/sex. The interaction terms were only included in the model if the p-values were <0.05. In model 1.C the variable parental history was categorized as no parental history of AF, one parent with AF and two parents with AF. In model 1.D, we investigated the association of parental history of LAF with time to first recurrence of AF in proband cases.

In order to further evaluate the results, we also investigated time from first until second recurrence of AF (model 1.E), as well as time from diagnosis of LAF until second recurrence of AF (model 1.F, n=7,370). We additionally investigated time to first recurrence of AF in individuals who did not experience any other CVD outcome during the 10-year follow-up period (model 1.G, n=9,071).

In the second analysis, a sibling analysis, we examined all LAF proband cases with at least one sibling living in Sweden sometime between 1987 and 2009 (n=20,373). In model 2.A, sibling history of AF was included as a covariate (yes/no), and in model 2.B we also included sex, age at diagnosis of LAF (centered at the mean value), and terms for the interactions between sibling history of AF and age/sex. The interaction terms were only included in the model if the p-values were <0.05. We also adjusted both models for number of siblings to the proband case (not reported in the tables).

In the third analysis, we merged datasets I and II and only analyzed individuals who were included in both datasets (13,525 cases). In model 3.A, sibling history of AF and parental history of AF were included as covariates (yes/no), and in model 3.B we also included sex, age at diagnosis of LAF (age at LAF), terms for the interactions between sibling history of AF and parental history of AF and terms for the interactions between sibling history of AF/parental history of AF and age/sex. The interaction terms were only included in the model if the p-values were <0.05.

In order to take into account the non-independence of observations from the same family, we used a robust sandwich estimator in all models [34,35]. We present hazard ratios (HRs) and the corresponding 95% CIs [39]. The proportional hazards assumption was fulfilled for the variables of interest.

In order to investigate familial transmission of first time LAF hospitalization we used a case-cohort approach to determine familial risks with odds ratios (ORs) [34,35]. We conducted two main analyses: proband-sibling and proband-parent. In these analyses, we studied all LAF proband-relative pairs that could be matched to five control pairs in the Swedish population. For example, in the proband-sibling analysis we selected all sibling pairs for which at least one sibling was diagnosed with LAF and matched each of them to five control pairs. The control pairs were chosen randomly from individuals who lived in Sweden at the time of the probands’ diagnosis of LAF and comprised pairs of individuals who were not diagnosed with LAF or AF prior to the time of the proband’s diagnosis of LAF. Furthermore, both individuals in the control pair also had to have lived in Sweden sometime during the period 1987–2009. Control pairs were matched based on year of birth, sex, country of birth and level of education (the year before the date of diagnosis). Analyses were conducted by conditional logistic regression [34,35]. As an example, in the proband-sibling analysis, AF in sibling (yes/no) was used as the independent variable. We present odds ratios (ORs) and the corresponding 95% CIs, according to previous studies of familial risks [34,35]. As a proband could be included several times, we adjusted for non-independence by using a robust sandwich estimator [34,35]. In all analyses, less than 1% of the proband pairs could not be matched to five controls and were excluded from the analysis.

All calculations were performed using SAS version 9.3.

## Results

There were 16,160 individuals with LAF in families in which both parents were alive sometime during the study period (Table 1). 45.6% of them had recurrent hospitalization for AF. 26.9% of individuals with recurrence had a parent with AF, compared to 21.6% of those without recurrence. There were 20,373 individuals with LAF in families in which at least one sibling was alive sometime during the study period. 48.6% of them had recurrent hospitalization for AF. 11.6% of the individuals with recurrence had at least one sibling diagnosed with AF during the study period. The corresponding number for non-recurrent cases was 7.7%.

**Table 1:** Descriptive statistics for individuals with lone atrial fibrillation (LAF) in the Swedish population (1987–2009)

	**Individuals diagnosed with LAF and with both parents living in Sweden sometime during 1987–2009 (n=16,160)**		**Individuals diagnosed with LAF and with at least one sibling living in Sweden sometime during 1987–2009 (n=20,373)**	
	**Recurrence**	**No Recurrence**	**Recurrence**	**No Recurrence**
N	7,375	8,785	9,909	10,464
AF parents	26.9%	21.6%		
LAF parents	2.7%	2.5%		
AF siblings			11.6%	7.7%
Men	79.0%	75.8%	77.2%	74.1%
Age at diagnosis of LAF, mean (SD)	48.3 (10.1)	46.3 (11.1)	50.5 (9.0)	48.6 (10.7)

**Figure 1:**
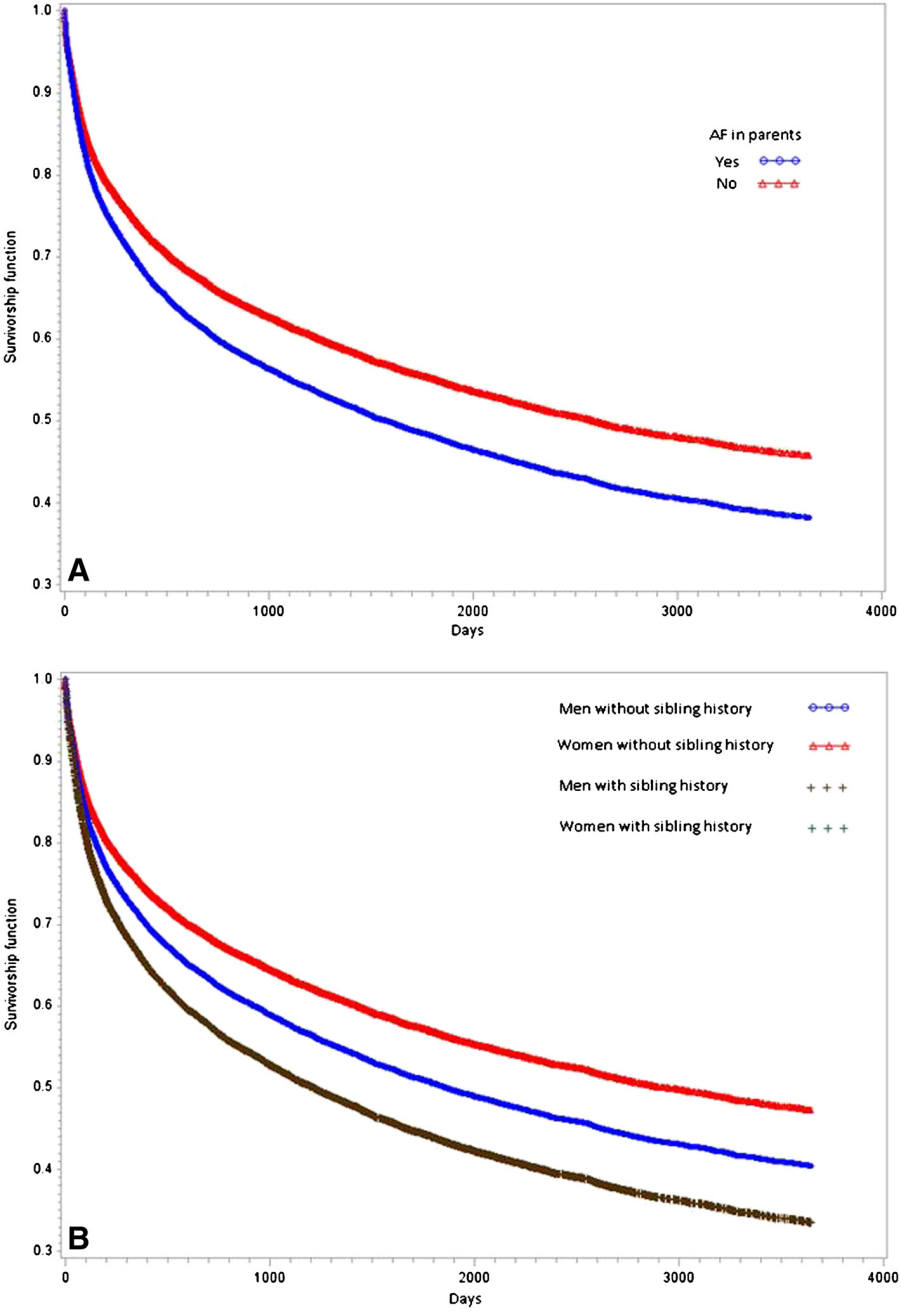
**A (left) Survivorship function for model 1.A, i.e. parental history of AF (yes/no). B** (right) Survivorship function for model 2.A, ie. sibling history of AF (yes/no). Note that the survivorship function for men with sibling history of AF overlaps with the survivorship function for women with sibling history of AF.

Table 2, model 1.A shows that proband cases with parents diagnosed with AF had a 1.23 times higher hazard (95% CI 1.17-1.30) for recurrent AF hospitalization than proband cases without parental history. This number was not attenuated when age and sex were included in the model (Table 2, model 1B). However, there seemed to be an interaction between age at diagnosis of AF among parents (HR 0.99, 95% CI 0.99-0.99), indicating a decreased association of parental AF on recurrent AF the older the case. Model 1.C indicates that the HR was higher for proband cases with two affected parents (HR 1.65, 95% CI 1.44-1.90) than for proband cases with only one affected parent (HR 1.16, 95% CI 1.10-1.23). Model 1.D shows that proband cases with parents diagnosed with LAF had a 1.42-fold higher hazard rate for recurrent AF hospitalization. While model 1.E shows that parental history is less important when investigating time between second and third episodes of AF, model 1.F shows that the HR for parental history is similar when investigating time from first to second episode of AF and time from first to third episode of AF. The HR in model 1.G (in which all cases with a CVD diagnosis in the 10 years after first AF hospitalization were excluded) is similar to the HR in model 1.A. This indicates that the results in model 1.A are not confounded by other CVD outcomes. Figure 1A shows that at the end of the follow-up period, approximately 60% of proband cases with parental history of AF had recurrent hospitalization for AF; the corresponding number for proband cases without parental history was 50%.

**Table 2:** Results from Cox proportional hazard models for individuals with LAF in the Swedish population (1987–2009): parent-offspring analysis (n=16,160)

**Variable**	**Model 1.A**	**Model 1.B**	**Model 1.C**	**Model 1.D**
	**HR (95% CI)**	**HR (95% CI)**	**HR (95% CI)**	**HR (95% CI)**
AF in parent(s) (yes/no)	1.23 (1.17-1.30)	1.22 (1.15-1.28)		
AF in 1 parent			1.16 (1.10-1.23)	
AF in 2 parents			1.65 (1.44-1.90)	
LAF in parent(s) (yes/no)				1.42 (1.23-1.63)
Sex (Reference: females)		1.20 (1.13-1.27)	1.20 (1.13-1.27)	1.20 (1.13-1.27)
Age at diagnosis of LAF (yearly increase)		1.02 (1.02-1.02)	1.02 (1.02-1.02)	1.02 (1.02-1.02)
Age at diagnosis of AF in parent(s)		0.99 (0.99-0.99)		
	**Model 1.E**	**Model 1.F**	**Model 1.G**	
	**HR (95% CI)**	**HR (95% CI)**	**HR (95% CI)**	
AF in parent(s)	1.07 (1.00-1.14)	1.22 (1.15-1.31)	1.23 (1.15-1.31)	
Sex (Reference: females)	1.05 (0.97-1.13)	1.22 (1.14-1.31)	1.22 (1.14-1.31)	
Age at diagnosis of LAF (yearly increase)	1.00 (1.00-1.01)	1.02 (1.02-1.02)	1.02 (1.02-1.02)	

Model 1.A: HR of parental history of AF. Model 1B: also included sex, age at diagnosis of LAF (centered at the mean value), and terms for the interaction between parental history of AF and age/sex. The interaction terms were only included in the model if the p-values were <0.05. Model 1C: the variable parental history was categorized as no parental history of AF, one parent with AF and two parents with AF. Model 1D: the association of parental history of LAF with time to first recurrence of AF in proband cases in proband cases was determined. Model 1E: time from first until second recurrence of AF was used to calculate the familial HR. Model 1F: time from diagnosis of LAF until second recurrence of AF was used. Model 1G: time to first recurrence of AF in individuals who did not experience any other CVD outcome during the 10-year follow-up period was used to calculate HR.

Table 3, model 2.A shows that proband cases with at least one sibling diagnosed with AF had a 1.30-fold higher hazard rate (95% CI 1.22-1.38) for recurrent AF hospitalization than proband cases without sibling history. However, model 2.B shows that the risk of LAF associated with sibling history differed between men and women (HR for interaction term 0.83, 95% CI 0.72-0.95). This indicates that even though men had a higher risk of recurrent AF hospitalization (HR 1.21), the effect of AF among siblings was more important for women than for men. Figure 1B shows the survivorship function from model 2.B, indicating that at the end of the follow-up period 65% of individuals with at least one sibling with AF had recurrent hospitalization for AF. This number was the same for both men and women. Model 3.A shows that the HR was similar for parental and sibling history of AF (HR 1.21 and 1.28, respectively).

**Table 3:** Results from Cox proportional hazard models for individuals with LAF in the Swedish population (1987–2009): sibling analysis (n=20,373) and parent-sibling analysis (n=13,525)

	**Model 2.A**	**Model 2.B**	**Model 3.A**	**Model 3.B**
	**HR (95% CI)**	**HR (95% CI)**	**HR (95% CI)**	**HR (95% CI)**
AF in sibling(s) (yes/no)	1.30 (1.22-1.38)	1.46 (1.30-1.65)	1.28 (1.18-1.39)	1.48 (1.24-1.75)
AF in parent(s)			1.21 (1.14-1.28)	1.16 (1.10-1.23)
Sex (Reference: females)		1.21 (1.15-1.27)		1.26 (1.18-1.34)
Age at diagnosis of AF (yearly increase)		1.02 (1.02-1.02)		1.02 (1.02-1.02)
AF in Sibling (age at diagnosis of AF)		0.99 (0.98-0.99)		
AF in sibling (sex)		0.83 (0.72-0.95)		0.76 (0.63-0.93)
AF in parent(s) (age at diagnosis of AF)				0.99 (0.98-0.997)

Model 2.A: sibling history of AF was included as a covariate (yes/no). Model 2.B: we also included sex, age at diagnosis of LAF (centered at the mean value), and terms for the interactions between sibling history of AF and age/sex. The interaction terms were only included in the model if the p-values were <0.05. Both model 2A and 2B were adjusted for the number of siblings to the proband case. Model 3.A, sibling history of AF and parental history of AF were included as covariates (yes/no). Model 3.B: included sex, age at diagnosis of LAF (age at LAF), terms for the interactions between sibling history of AF and parental history of AF and terms for the interactions between sibling history of AF/parental history of AF and age/sex. The interaction terms were only included in the model if the p-values were <0.05.

Familial risk of first time LAF hospitalization was determined for comparison with recurrent risk only. Table 4 shows the results of the case-cohort analysis. 14.2% of the proband cases had a parent diagnosed with AF. The OR for the parent-offspring analysis was 2.08 (95% CI 2.02-2.15), which can be interpreted as a 2-fold increase in the odds of AF in the parents of proband cases diagnosed with LAF compared to the parents to controls. The sibling analysis revealed that 5.3% of the proband cases had a sibling diagnosed with AF. The OR was 3.23 (95% CI 3.08-3.39), indicating more than 3-fold higher odds of AF siblings of individuals diagnosed with LAF compared to the siblings of controls.

**Table 4:** Familial risk of first time LAF hospitalization in individuals with parental or sibling history of AF

**Relationship to proband**	**Number of pairs**	**Concordant pairs**	OR (95% CI)*
Sibling	40,818	2,167 (5.3%)	3.23 (3.08-3.39)
Parent	34,657	4,906 (14.2%)	2.08 (2.02-2.15)

Results from conditional logistic regression analysis of individuals with LAF in the Swedish population (1987–2009).

*OR ratio was determined according to previous studies of familial risks [34,35].

## Discussion

The present study is, to our knowledge, the first nationwide study to estimate the familial risk of recurrent hospitalization for LAF. Previous studies have only investigated the familial risk of a first AF event [6-15]. The present study found similar familial risks for first time LAF event to those reported previously [6-15]. The familial risk for recurrent LAF hospitalization was lower than the risk for first time LAF hospitalization, suggesting that familial and possibly genetic factors are more important for first time LAF hospitalization than recurrent LAF hospitalization. Our findings indicate that family history of AF is a new risk factor for recurrent hospitalization for LAF. The higher risk for recurrent LAF hospitalization among multiplex families (i.e. two or more affected relatives) may be genetic although non-genetic familial factors cannot be ruled out. There was an interaction between age and family history, with family history having a weaker effect on AF hospitalization risk in older age groups, which further indicates a genetic contribution. Identifying genetic risk factors for AF may have important prognostic value regarding recurrence risk. The mechanism behind the observed familial recurrent LAF hospitalization risk might be genetic or non-genetic. For instance, patients with familial LAF might have a higher AF awareness and possibly have a lower threshold for seeking health care. However, several genetic variants have been linked to risk of AF [16-25]. It is possible that these variants may also predispose individuals to an increased risk of recurrent hospitalization for LAF. Moreover, the dissected underlying familial risk factors (genetic or nongenetic) are likely to have a magnitude that is stronger than the familial risk itself [40,41]. For instance, it has been estimated that 10 additive alleles, each with a genotype relative risk of 2.0 and an allele frequency of 0.1, will only explain a familial risk of 1.06 [41]. If multiplicative gene-gene interactions are present, the same 10 alleles will still only explain a familial risk of 1.2 [41].

Interestingly, there appears to be an interaction between sex and family history, with family history of AF having a greater effect in women than in men in the context of risk of recurrent hospitalization for AF. This suggests that familial and possibly genetic factors might be relatively more important in women than in men regarding recurrent risk of hospitalization for LAF. In individuals without family history of AF, male sex was a risk factor for recurrent LAF. This is opposite to a study that found female sex to be associated with recurrence of AF [27]. However, the patients in that study were older and had cardiovascular comorbidities, which may explain the divergent results. We also investigated whether family history of AF is a risk factor for a third recurrent LAF hospitalization. No such association was found. This may be due to that having a personal history of two or more AF is a strong risk factor in itself for recurrent AF [29].

The present study has a number of strengths. These include complete nationwide coverage in a country with high medical standards and medical diagnosis of patients by specialists during examinations in hospitals. In addition, the results were not affected by recall bias because both the probands and cases were medically diagnosed. Importantly, the Multigeneration Register is a validated source that has been proved to be reliable in the study of many familial diseases [33-37].

In order to exclude cardiovascular confounders we excluded all LAF patients who developed CVD during the 10 year follow up period after first LAF hospitalization (Table 2, model 1G). The HR for recurrent LAF hospitalization was similar, which suggests that the results are not confounded by other CVD outcomes during follow up.

The present study has also a number of limitations. The most important limitation is that we do not know whether recurrent LAF hospitalization is due to a new episode of paroxysmal AF or to the same persistent episode of AF. Thus, the results could reflect an increased risk of recurrent paroxysmal AF or symptomatic persistent AF in familial cases. The increased risk for recurrent hospitalization persisted during the whole follow up period (10 years after first hospitalization), which suggests that our results mainly reflect an increased risk of recurrent paroxysmal AF. Early recurrence could sometimes be due to admittance for elective cardioversion of persistent AF after at least three weeks of treatment with anticoagulation. However, normally elective cardioversion is performed in outpatients.

Longitudinal registers are subject to missing data before the date the register started. The Swedish Hospital Discharge Register only contains complete data for the period since 1987. However, such losses would be similar for both cases and controls and would not affect the estimates of familial aggregation (i.e. the HR). This is most likely a source of non-differential bias regarding familial risk estimates.

Another potential limitation is that we do not have access to the methods used for objective diagnosis. However, the Swedish Hospital Discharge Register has high validity, especially for cardiovascular disorders such as AF (97%), stroke and myocardial infarction (approximately 95%) [37,38]. However, hypertension is not specifically validated and it is possible that the prevalence of this diagnoses is underestimated. It is therefore possible that some non-LAF cases have been included in the LAF group. This may have underestimated the familial recurrence of LAF, due to dilution of LAF cases with non-LAF cases.

While not all patients may seek help for AF, affordability of healthcare is probably not a selective factor in Sweden because of equal access to primary and hospital care. However, the likelihood of seeking medical advice might be of importance. It is possible that patients with familial LAF might have a higher AF awareness and possibly have a lower threshold for seeking health care. Thus, familial non-genetic factors might also be of importance.

## Conclusions

The present study demonstrates that family history of AF is a risk factor for recurrent hospitalization for LAF in Swedish patients. Risk of recurrent hospitalization for LAF was especially high in multiplex families and younger individuals, which suggests a genetic contribution. However, the familial risk for recurrent LAF hospitalization was lower than the risk for first time LAF hospitalization, suggesting that familial factors are more important for first time LAF hospitalization than recurrent LAF hospitalization.

## Abbreviations

AF: Atrial fibrillation; LAF: Lone atrial fibrillation; CI: Confidence interval; HR: Hazard ratio; OR: Odds ratio; ICD: International Classification of Diseases.

## Competing interests

The authors declare that they have no competing interest.

## Authors’ contributions

All authors contributed to the conception and design of the study; JS and KS contributed to the acquisition of data; all authors contributed to the analysis and interpretation of data; BZ drafted the manuscript; and all authors revised it critically and approved the final version. All authors had full access to all of the data (including statistical reports and tables) and take responsibility for the integrity of the data and the accuracy of their analysis.

## Pre-publication history

The pre-publication history for this paper can be accessed here:

http://www.biomedcentral.com/1471-2261/12/121/prepub

## References

[B1] Estes NA 3rd Sacco RL Al-Khatib SM Ellinor PT Bezanson J Alonso A Antzelevitch C Brockman RG Chen PS Chugh SS Curtis AB DiMarco JP Ellenbogen KA Epstein AE Ezekowitz MD Fayad P Gage BF Go AS Hlatky MA Hylek EM Jerosch-Herold M Konstam MA Lee R Packer DL Po SS Prystowsky EN Redline S Rosenberg Y Van Wagoner DR Wood KA American Heart Association atrial fibrillation research summit: a conference report from the American Heart Association Circulation 2011 124 363 372 10.1161/CIR.0b013e318224b037 21709057 PMC3144149

[B2] Lip GY Tse HF Lane DA Atrial fibrillation Lancet 2012 379 648 661 10.1016/S0140-6736(11)61514-6 22166900

[B3] Schoonderwoerd BA Smit MD Pen L Van Gelder IC New risk factors for atrial fibrillation: causes of ‘not-so-lone atrial fibrillation’ Europace 2008 10 668 673 10.1093/europace/eun124 18480076

[B4] Evans W Swann P Lone auricular fibrillation Br Heart J 1954 16 189 194 10.1136/hrt.16.2.189 13160271 PMC479515

[B5] Potpara TS Lip GY Lone atrial fibrillation: what is known and what is to come Int J Clin Pract 2011 65 446 457 10.1111/j.1742-1241.2010.02618.x 21219558

[B6] Wolf L Familial auricular fibrillation N Engl J Med 1943 229 396 397 10.1056/NEJM194309022291002

[B7] Darbar D Herron KJ Ballew JD Jahangir A Gersh BJ Shen WK Hammill SC Packer DL Olson TM Familial atrial fibrillation is a genetically heterogeneous disorder J Am Coll Cardiol 2003 41 2185 2192 10.1016/S0735-1097(03)00465-0 12821245

[B8] Fox CS Parise H D’Agostino RB Sr Lloyd-Jones DM Vasan RS Wang TJ Levy D Wolf PA Benjamin EJ Parental atrial fibrillation as a risk factor for atrial fibrillation in offspring JAMA 2004 291 2851 2855 10.1001/jama.291.23.2851 15199036

[B9] Ellinor PT Yoerger DM Ruskin JN MacRae CA Familial aggregation in lone atrial fibrillation Hum Genet 2005 118 179 184 10.1007/s00439-005-0034-8 16133178

[B10] Arnar DO Thorvaldsson S Manolio TA Thorgeirsson G Kristjansson K Hakonarson H Stefansson K Familial aggregation of atrial fibrillation in Iceland Eur Heart J 2006 27 708 712 10.1093/eurheartj/ehi727 16428254

[B11] Marcus GM Smith LM Vittinghoff E Tseng ZH Badhwar N Lee BK Lee RJ Scheinman MM Olgin JE A first-degree family history in lone atrial fibrillation patients Hear Rhythm 2008 5 826 830 10.1016/j.hrthm.2008.02.016 PMC2474569 18468961

[B12] Chen LY Herron KJ Tai BC Olson TM Lone atrial fibrillation: influence of familial disease on gender predilection J Cardiovasc Electrophysiol 2008 19 802 806 10.1111/j.1540-8167.2008.01126.x 18363686 PMC2562911

[B13] Christophersen IE Ravn LS Budtz-Joergensen E Skytthe A Haunsoe S Svendsen JH Christensen K Familial aggregation of atrial fibrillation: a study in Danish twins Circ Arrhythm Electrophysiol 2009 2 378 383 10.1161/CIRCEP.108.786665 19808493 PMC2760022

[B14] Yang YQ Zhang XL Wang XH Tan HW Shi HF Fang WY Liu X Familial aggregation of lone atrial fibrillation in the Chinese population Intern Med 2010 49 2385 2391 10.2169/internalmedicine.49.4130 21088338

[B15] Lubitz SA Yin X Fontes JD Magnani JW Rienstra M Pai M Villalon ML Vasan RS Pencina MJ Levy D Larson MG Ellinor PT Benjamin EJ Association between familial atrial fibrillation and risk of new-onset atrial fibrillation JAMA 2010 304 2263 2269 10.1001/jama.2010.1690 21076174 PMC3073054

[B16] Brugada R Tapscott T Czernuszewicz G Marian AJ Iglesias A Mont L Brugada J Girona J Domingo A Bachinski LL Roberts R Identification of a genetic locus for familial atrial fibrillation N Engl J Med 1997 336 905 911 10.1056/NEJM199703273361302 9070470

[B17] Gudbjartsson DF Arnar DO Helgadottir A Gretarsdottir S Holm H Sigurdsson A Jonasdottir A Baker A Thorleifsson G Kristjansson K Palsson A Blondal T Sulem P Backman VM Hardarson GA Palsdottir E Helgason A Sigurjonsdottir R Sverrisson JT Kostulas K Ng MC Baum L So WY Wong KS Chan JC Furie KL Greenberg SM Sale M Kelly P MacRae CA Variants conferring risk of atrial fibrillation on chromosome 4q25 Nature 2007 448 353 357 10.1038/nature06007 17603472

[B18] Gudbjartsson DF Holm H Gretarsdottir S Thorleifsson G Walters GB Thorgeirsson G Gulcher J Mathiesen EB Njølstad I Nyrnes A Wilsgaard T Hald EM Hveem K Stoltenberg C Kucera G Stubblefield T Carter S Roden D Ng MC Baum L So WY Wong KS Chan JC Gieger C Wichmann HE Gschwendtner A Dichgans M Kuhlenbäumer G Berger K Ringelstein EB A sequence variant in ZFHX3 on 16q22 associates with atrial fibrillation and ischemic stroke Nat Genet 2009 41 876 878 10.1038/ng.417 19597491 PMC2740741

[B19] Benjamin EJ Rice KM Arking DE Pfeufer A van Noord C Smith AV Schnabel RB Bis JC Boerwinkle E Sinner MF Dehghan A Lubitz SA D'Agostino RB Sr Lumley T Ehret GB Heeringa J Aspelund T Newton-Cheh C Larson MG Marciante KD Soliman EZ Rivadeneira F Wang TJ Eiríksdottir G Levy D Psaty BM Li M Chamberlain AM Hofman A Vasan RS Variants in ZFHX3 are associated with atrial fibrillation in individuals of European ancestry Nat Genet 2009 41 879 881 10.1038/ng.416 19597492 PMC2761746

[B20] Ellinor PT Lunetta KL Glazer NL Pfeufer A Alonso A Chung MK Sinner MF de Bakker PI Mueller M Lubitz SA Fox E Darbar D Smith NL Smith JD Schnabel RB Soliman EZ Rice KM Van Wagoner DR Beckmann BM van Noord C Wang K Ehret GB Rotter JI Hazen SL Steinbeck G Smith AV Launer LJ Harris TB Makino S Nelis M Common variants in KCNN3 are associated with lone atrial fibrillation Nat Genet 2010 42 240 244 10.1038/ng.537 20173747 PMC2871387

[B21] Wakili R Voigt N Kääb S Dobrev D Nattel S Recent advances in the molecular pathophysiology of atrial fibrillation J Clin Invest 2011 121 2955 2968 10.1172/JCI46315 21804195 PMC3148739

[B22] Parvez B Darbar D The “missing link” in atrial fibrillation heritability J Electrocardiol 2011 44 641 644 10.1016/j.jelectrocard.2011.07.027 21924735 PMC3200486

[B23] Olesen MS Jespersen T Nielsen JB Liang B Møller DV Hedley P Christiansen M Varró A Olesen SP Haunsø S Schmitt N Svendsen JH Mutations in sodium channel β-subunit SCN3B are associated with early-onset lone atrial fibrillation Cardiovasc Res 2011 89 786 793 10.1093/cvr/cvq348 21051419

[B24] Watanabe H Darbar D Kaiser DW Jiramongkolchai K Chopra S Donahue BS Kannankeril PJ Roden DM Mutations in sodium channel beta1- and beta2-subunits associated with atrial fibrillation Circ Arrhythm Electrophysiol 2009 2 268 275 10.1161/CIRCEP.108.779181 19808477 PMC2727725

[B25] Ellinor PT Lunetta KL Albert CM Glazer NL Ritchie MD Smith AV Arking DE Müller-Nurasyid M Krijthe BP Lubitz SA Bis JC Chung MK Dörr M Ozaki K Roberts JD Smith JG Pfeufer A Sinner MF Lohman K Ding J Smith NL Smith JD Rienstra M Rice KM Van Wagoner DR Magnani JW Wakili R Clauss S Rotter JI Steinbeck G Meta-analysis identifies six new susceptibility loci for atrial fibrillation Nat Genet 2012 44 670 675 10.1038/ng.2261 22544366 PMC3366038

[B26] Liu T Li G Li L Korantzopoulos P Association between C-reactive protein and recurrence of atrial fibrillation after successful electrical cardioversion: a meta-analysis J Am Coll Cardiol 2007 49 1642 1648 10.1016/j.jacc.2006.12.042 17433956

[B27] Gurevitz OT Varadachari CJ Ammash NM Malouf JF Rosales AG Herges RM Bruce CJ Somers VK Hammill SC Gersh BJ Friedman PA The effect of patient sex on recurrence of atrial fibrillation following successful direct current cardioversion Am Heart J 2006 152 155 9 13 10.1016/j.ahj.2006.04.030 16824847

[B28] Guglin M Maradia K Chen R Curtis AB Relation of obesity to recurrence rate and burden of atrial fibrillation Am J Cardiol 2011 107 579 582 10.1016/j.amjcard.2010.10.018 21195377

[B29] Disertori M Lombardi F Barlera S Latini R Maggioni AP Zeni P Di Pasquale G Cosmi F Franzosi MG GISSI-AF Investigators Clinical predictors of atrial fibrillation recurrence in the Gruppo Italiano per lo Studio della Sopravvivenza nell'Infarto Miocardico-Atrial Fibrillation (GISSI-AF) trial Am Heart J 2010 159 857 863 10.1016/j.ahj.2010.02.016 20435196

[B30] Schmidt M Rieber J Daccarett M Marschang H Sinha AM Biggar P Jung P Ketteler M Brachmann J Rittger H Relation of recurrence of atrial fibrillation after successful cardioversion to renal function Am J Cardiol 2010 105 368 372 10.1016/j.amjcard.2009.09.037 20102950

[B31] Latini R Masson S Pirelli S Barlera S Pulitano G Carbonieri E Gulizia M Vago T Favero C Zdunek D Struck J Staszewsky L Maggioni AP Franzosi MG Disertori M on the behalf of the GISSI-AF Investigators Circulating cardiovascular biomarkers in recurrent atrial fibrillation: data from the GISSI-atrial fibrillation trial J Intern Med 2011 269 160 171 10.1111/j.1365-2796.2010.02287.x 20964739

[B32] Tang Y Yang H Qiu J Relationship between brain natriuretic peptide and recurrence of atrial fibrillation after successful electrical cardioversion: a meta-analysis J Int Med Res 2011 39 1618 1624 22117962 10.1177/147323001103900504

[B33] Rosen M Hakulinen T Ahrens W, Pigeot I Use of disease registers Handbook of epidemiology 2005 Springer-Verlag: Berlin 231 252

[B34] Lichtenstein P Yip BH Björk C Pawitan Y Cannon TD Sullivan PF Hultman CH Common genetics determinants of schizophrenia and bipolar disorders in Swedish families: a population-based study Lancet 2009 373 234 239 10.1016/S0140-6736(09)60072-6 19150704 PMC3879718

[B35] Zöller B Li X Ohlsson H Sundquist J Sundquist K Venous thromboembolism does not share strong familial susceptibility with ischemic stroke: a nationwide family study in Sweden Circ Cardiovasc Genet 2011 4 484 490 10.1161/CIRCGENETICS.111.959882 21880672

[B36] Zöller B Li X Sundquist J Sundquist K Age- and gender-specific familial risks for venous thromboembolism: a nationwide epidemiological study based on hospitalizations in Sweden Circulation 2011 124 1012 1020 10.1161/CIRCULATIONAHA.110.965020 21824919

[B37] Ludvigsson JF Andersson E Ekbom A Feychting M Kim JL Reuterwall C Heurgren M Olausson PO External review and validation of the Swedish national inpatient register BMC Publ Health 2011 11 450 10.1186/1471-2458-11-450 PMC3142234 21658213

[B38] Smith JG Platonov PG Hedblad B Engström G Melander O Atrial fibrillation in the Malmö Diet and Cancer study: a study of occurrence, risk factors and diagnostic validity Eur J Epidemiol 2010 25 95 102 10.1007/s10654-009-9404-1 19936945

[B39] Rothman K Greenland S Modern Epidemiology 2008 3 Philadelphia: Lippincott-Raven

[B40] Burton PR Tobin MD Hopper JL Key concepts in genetic epidemiology Lancet 2005 366 941 951 10.1016/S0140-6736(05)67322-9 16154023

[B41] Hemminki K Bermejo JL Constraints for genetic association studies imposed by attributable fraction and familial risks Carcinogenesis 2007 28 648 656 17012223 10.1093/carcin/bgl182

